# Surgical Advances in Paediatric Neuroblastoma

**DOI:** 10.3390/children9050663

**Published:** 2022-05-05

**Authors:** Giuseppe Martucciello

**Affiliations:** 1 Dipartimento di Neuroscienze, Riabilitazione, Oftalmologia, Genetica e Scienze Materno-Infantili (DiNOGMI), University of Genova, 16132 Genoa, Italy; giuseppemartucciello@gaslini.org; 2 Department of Paediatric Surgery, IRCCS Giannina Gaslini Children’s Hospital, 16147 Genoa, Italy

As an introduction to this “Special Issue” launched by *Children* (ISSN 2227-9067), I would firstly like to highlight those neoplasms should be labeled with the more appropriate term “Neuroblastomas” (NBs). This is to emphasize their very heterogeneous clinical and biological behavior.

To provide a historical insight, in 1974, Bolande firstly used the term “neurocristopathies” to name this constellation of various conditions and classified these neural crest diseases into two main forms:

i. “Simple neurocristopathies”, are characterized by a single pathologic process, which is generally unifocal and localized;
ii. “Neurocristopathic syndromes and complex neurocristopathies”, corresponding to multifocal and varied associations of simple neurocristopathies [1].

Their heterogeneous location and clinical presentation mainly originate from primordial neural crest cells, which migrate from the mantle layer of the developing spinal cord into the neck, the posterior mediastinum, the adrenal medulla, the retroperitoneal paraspinal ganglia, and the pelvic organ of Zuckerkandl (a chromaffin body derived from the neural crest site at the bifurcation of the aorta or at the origin of the inferior mesenteric artery) [2,3].

Over the past three decades, several biological and genetic signatures have been identified as fundamental prognostic factors to guide NB treatments. In fact, to date, patient medical and surgical treatments are highly individualized using well-established staging systems and therapeutic protocols [4,5,6].

However, the complexity of their biology and the high variability of their clinical behavior make this common group of pediatric malignancies a clinical and surgical challenge.

In light of this, the structure of this “Special Issue” has been designed to include a variety of studies addressing several clinical and surgical topics, together with the most recent cutting-edge innovations derived from basic science. Modern pediatric surgeons are highly aware that overall knowledge of NB etiopathology, biology, and genetics is required to properly approach these solid tumors. It is important to note that basic science is now offering several adjuncts to their surgical and clinical treatment. In the field of surgery, there have been significant drives toward the clinical translation of new technologies, devices, and intra-operative techniques to enable surgeons to perform safer and more radical excisions [7]. For all these reasons, I recommend young registrars interested in pediatric oncology to get involved in preclinical research by undertaking research fellowships in highly qualified research centers.

In this regard, the article “Molecular Genetics in Neuroblastoma Prognosis” [8] has been included in this “Special Issue”. It describes how, in recent years, many efforts have been made to identify the biological and genetic features of NBs in order to improve patient staging and treatment stratification. Their principal genetic signature and the most recent scientific advances in molecular genetics have been discussed, focusing on their impact on diagnosis, prognosis, and clinical management. The advent of the genomic era has provided significant improvements in NB genetic characterization. The opportunity to analyze big datasets of patient data has surely provided essential information to better understand the genetic patterns involved in NB tumorigenesis and progression.

NB is the most common extra-cranial solid tumor in children, representing 8–10% of all childhood tumors and approximately 15% of all cancer-related deaths in the pediatric population [9]. The overall survival of children with high-risk diseases is around 40–50%, despite the aggressive treatment protocols consisting of intensive chemotherapy, surgery, radiation therapy, and hematopoietic stem cell transplantation [10,11].

These protocols highly rely on the possibility of studying the expression of biological markers of tumor progression and treatment response. Thus, studying the biological tumor profile is mandatory, and it is usually performed on the primary tumor tissue. NB sampling has, therefore, become essential to characterize their molecular signature. Unfortunately, however, many NB tissue biopsies are complicated by the lack of neoplastic cells/tissue. Needle biopsies are limited by several factors:

i. The difficulty of obtaining an adequate amount of tissue;
ii. The invasive nature of this technique, which prevents its repeatability;
iii. The impossibility of capturing the tumor histological heterogeneity.

All these reasons justify the strong interest of the scientific community in the discovery of new alternatives and more easily applicable techniques to obtain biological material (such as liquid biopsies).

The second article included in this “Special Issue” deals with the perspectives of preclinical research and is entitled “Novel Treatments and Technologies Applied to the Cure of Neuroblastoma” [12]. This review article describes ongoing research efforts to increase NBs’ cellular and molecular biology knowledge to translate essential findings into novel treatment strategies. In fact, surgery represents a cornerstone within the multimodal treatment of NB, and the literature supports the idea that a gross total resection is associated with better survival outcomes. However, performing a radical NB excision is particularly demanding due to the strict adhesion of the tumor to the major abdominal blood vessels and nerves and the extensive tumor fibrosis resulting from neoadjuvant chemotherapy. By facilitating the tumor’s discrimination from the surrounding normal tissue, fluorescence-guided surgery (FGS) has the potential to lower the risk of intra-operative complications and increase the survival rate of childhood tumors [13].

Great emphasis in this “Special Issue” is given to the chapter that compares different methods of NB biopsy. In fact, molecular phenotype analyses highly rely on the choice of the most informative biopsy techniques. In “The Role of Biopsy in the Workup of Patients with Neuroblastoma: Comparison of the Incidence of Surgical Complications and the Diagnostic Reliability of Diverse Techniques” [14], the authors describe their unicentric experience with four different techniques adopted for NB sampling:

i. Open incisional biopsy;
ii. Minimally invasive thoracoscopic/laparoscopic incisional biopsy;
iii. Ultrasound-guided core needle biopsy;
iv. Laparoscopic-assisted core needle biopsy.

The benefits of each technique, as well as their drawbacks, are analyzed.

In the surgical section of this “Special Issue”, the authors focus on different surgical accesses to resect NBs, aiming to perform a complete tumor excision with minimum surgical risks. These aspects are investigated in two studies, namely, “Surgical Approaches to Neuroblastoma: Review of the Operative Techniques” [15] and “The Cervico-Parasternal Thoracotomy (CPT): A New Surgical Approach for the Resection of Cervicothoracic Neuroblastomas” [16]. In more detail, three innovative incisional techniques are described in depth: the Cervico-Parasternal Thoracotomy (CPT) [16]; the ThoracoPhrenoLaparotomic resection (TPL) [17]; and the complete Posterior Sagittal Anorectal Mobilization (PSAM), which follows the same principles of the Posterior Sagittal AnoRectoPlasty (PSARP) firstly described by deVries and Peña [18,19].

Without claiming to be a state-of-the-art in pediatric NBs, this “Special Issue” represents an interesting insight into NBs. It collects a variety of medical and surgical perspectives on NB diagnosis and treatment, together with the description of the most innovative topics in preclinical research.

I am confident that all professionals and researchers involved in NB treatment will find this “Special Issue” particularly interesting. The ultimate aim is to improve the understanding of NBs, patient survival, and quality of life.
